# Diagnosis of alcohol misuse and alcoholic liver disease among patients in the medical emergency admission service of a large urban hospital in Sub-Saharan Africa; a cross sectional study

**DOI:** 10.11604/pamj.2013.15.23.2040

**Published:** 2013-05-13

**Authors:** Christopher Kenneth Opio, Emmanuel Seremba, Ponciano Ocama, Rejani Lalitha, Magid Kagimu, William Martens Lee

**Affiliations:** 1 Department of Medicine, Makerere University College of Health Sciences P.O.Box 7072, Kampala, Uganda; 2 Digestive and Liver Diseases, University of Texas Southwestern Medical School

**Keywords:** Alcohol use, alcohol misuse, alcoholic liver disease, aspartate aminotransferase, alanine aminotransferase, CAGE questionnaire, De Ritis ratio

## Abstract

**Introduction:**

Uganda is among the top ten consumers of alcohol worldwide though there is little data on alcohol related liver disease. We describe alcohol use, alcohol misuse, and alcoholic liver disease among adults at the emergency admission service of a large urban hospital in Uganda.

**Methods:**

All adults who consented were prospectively evaluated for alcohol use by inquiry and alcohol misuse by the “Cutting down, Annoyance, Guilt and Eye-opener- CAGE” questionnaire. Alcohol related hepatocellular liver injury was assessed using aspartate aminotransferase, and alanine aminotransferase levels. A combination of CAGE score ≥2 and De Ritis ratio ≥2 defined alcoholic liver disease (ALD). Human Immunodeficiency Virus (HIV), and viral hepatitis B and C serologies were evaluated in all the patients. Descriptive and inferential statistics were generated to answer our research questions.

**Results:**

Three hundred and eighty individuals consented and participated in the study. Among these, 46.8% acknowledged use of alcohol while 21% and 10% met the study definition of alcoholic misuse and alcoholic liver disease respectively. Both alcohol misuse and alcoholic liver disease was significantly associated (p-value ≤ 0.05) with male gender, region of origin, number of life time sexual partners and serum albumin below 3.5 mg/dl after univariate and multivariate analysis.

**Conclusion:**

Alcohol misuse and alcoholic liver disease is frequent in this medical emergency unit. Our study suggests a link between alcohol misuse or alcoholic liver disease and male gender, region of origin, number of sexual partners, and serum albumin below 3.5mg/dl.

## Introduction

Alcohol misuse is associated with poor health, disease and societal dysfunction [1, 2]. There is a clear relationship between per capita alcohol consumption and prevalence of alcoholic liver disease [3, 4]. Alcoholic liver disease, which is a liver disease because of alcohol consumption, is a common complication of alcohol misuse. It includes alcoholic fatty liver disease, alcoholic hepatitis and alcoholic cirrhosis. Diagnosis of alcoholic liver disease is usually made by documentation of excessive use of alcohol or alcohol misuse and clinical evidence of liver disease [5–7]. Recent alcohol use statistics indicate Uganda tops the world in consumption of alcohol at a total of 19.5 liters consumed per adult per annum [8]. However, there is no current data describing ALD in Uganda. We undertook a cross-sectional evaluation of all adult patients admitted at the emergency medical admission unit of Mulago hospital, the largest hospital in Uganda. We determined the prevalence of alcohol use, misuse and ALD, and factors associated with alcohol misuse and ALD.

## Methods

In 2005, within the months of January to March, we set out to initially determine the prevalence of HIV, Hepatitis B and C, and associated factors at the emergency admissions medical unit at Mulago hospital located in central Uganda. This emergency medical admissions unit admits an average of 40 adult medical patients per day. After obtaining informed consent, participants were consecutively recruited, interviewed, and blood draws for viral serologies and liver tests performed. Viral serology test results were performed using Cortez Rapid test^®^ (Cortez Diagnostics, Calabasas, CA) rapid tests. Aspartate aminotransferase (AST) and alanine aminotransferase (ALT) levels were determined by Cobas Integra 400 using Roche reagents on fresh serum samples with upper limit of 30 IU/L for both tests. The results of our findings concerning testing for viral hepatitis are reported elsewhere [9, 10]. In the same study, we also collected information on alcohol use, alcohol misuse, and ALD. From this data, we determined the extent of alcohol use, misuse and alcoholic liver disease as well as factors associated. Alcohol use was defined as a history of alcohol use (past or present). The CAGE questionnaire was used for measurement and diagnosis of alcohol misuse or abuse. CAGE questionnaire is a four item test with questions on Cutting down of alcohol intake (C), Annoyance at criticism about alcohol intake (A), Guilty feelings about alcohol intake (G) and use of Eye-openers morning drinking (E). Four affirmative responses were considered definite misuse or dependence), while two or three affirmative answers created a high level of suspicion for alcohol misuse/abuse [11]. Alcoholic liver disease was defined by the presence of alcohol misuse and *De Ritis ratio* ≥2:1 [12]. This information was entered into a data collection form and later transferred into excel spreadsheets. Following data preparation, the data was exported to Statistical Package STATA 10. Descriptive and inferential statistics were generated to answer our research objectives. We provided confidence intervals (CI), odds ratios (OR) where appropriate, and considered a p-value of ≤0.05 significant. For regression analysis, we included all variables that showed a trend to significance or were significantly associated with ALD at univariate analysis. We obtained ethical approval from Faculty of Medicine Research and Ethics Committee and the Uganda National Council of Science and Technology.

## Results

### Patient characteristics and alcohol misuse

**Figure 1:**
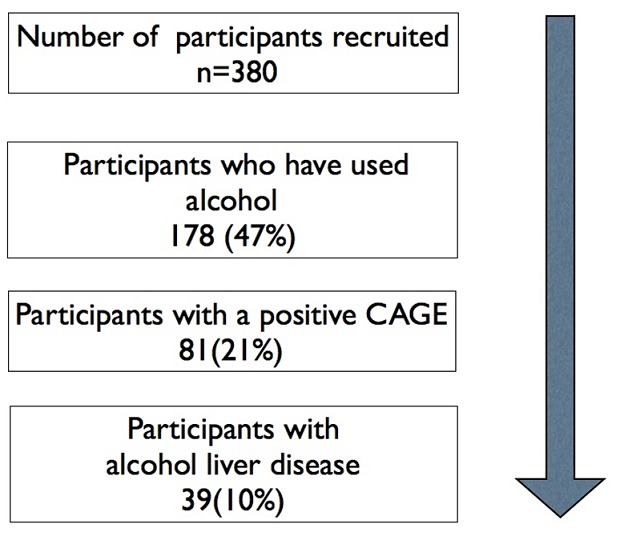
Proportion of participants with a history of alcohol use, alcohol misuse and alcoholic liver disease.

Over three months, we screened 420 adult patients and 380 patients met the study eligibility criteria that included written informed consent, over 17 years of age, and admission to the medical emergency ward. Most of the subjects were young; with a median age of 35 years (Inter Quartile Range- 21) and 55% were female. Among the 380 subjects, 178 (47%) reported use of alcohol and 81 (21%) had a positive CAGE score ≥2 (Figure 1). Of the 81 with a positive CAGE score, 62% had a high level of suspicion for alcohol misuse, while 38% met our criteria for definite alcohol misuse or dependence.

**Figure 2:**
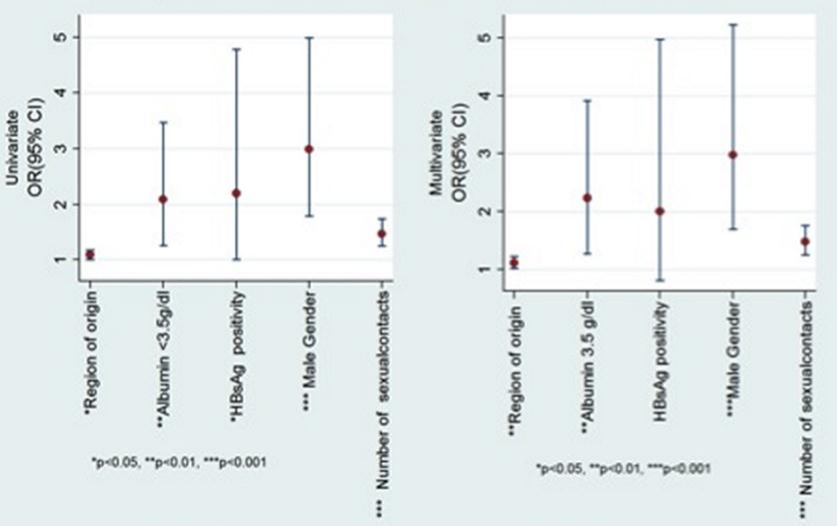
Analysis of alcohol misuse by various factors - Graphic summary of p-values, odds ratios, and 95% confidence intervals for univariate and multivariate analysis for gender, region of origin, number of life time sexual contacts, hepatitis B surface antigen status and serum albumin below 35g/l (3.5g/dl) among those with alcohol misuse compared to those without alcohol misuse.

Factors associated with alcohol misuse at univariate analysis included male gender (65% vs. 39%, p-value 0.001), originating from western and northern regions of Uganda (west- 22%vs 14% and north-10% vs. 7%, p-value 0.033), a history of more than 5 sexual partners (51% vs. 27%, p-value 0.001), a positive Hepatitis B surface antigen (14% vs. 7%, p value 0.045), and a serum albumin below 3.5 g/dl (63%vs 45%, p-value 0.004). However, only male gender (p-value Table 1 and Figure 2).

**Table 1:** Description and inference of clinical and laboratory characteristics of those with alcohol misuse compared to those without alcohol misuse.

Variable	Alcohol misuse	No Alcohol misuse	Statistical Inference
N, proportion	81 (21%)	299 (79%)	
Median age (years)	34	35	
Age IQR (years)	29- 48	25-48	
Male gender	53 (65%)	116 (39%)	p-value 0.001, 95% CI 1.8-5
**Region of origin**			p-value 0.033, 95% CI 1 -1.2
Central	47 (58%)	191 (64%)	
West	18(22%)	42(14%)	
North	8 (10%)	20 (7%)	
East	3(4%)	30(10%)	
Others	5(6%)	15 (5%)	
**Religion**			p-value 0.2, 95% CI 0.8 -1.0
Catholic	42(52%)	98 (33%)	
Protestant	22 (33%)	95 (32%)	
Muslim	6 (7%)	66 (22%)	
Pentecostal	2 (3%)	29 (10%)	
Others	4 (5%)	11 (4%)	
**Number of lifetime Sexual partners**			p-value 0.001, 95% CI 1.2 -1.7
1	10(13%)	90(35%)	
2	11(14%)	48(18%)	
3	11(14%)	39(15%)	
4	7 (9%)	12(5%)	
5 or more	41(51%)	71(27%)	
HBsAg sero +ve	11(14%)	20(7%)	p-value 0.045, 95% CI 1.0- 4.8
HIV sero +ve	43(53%)	140(47%)	**NS
Anti-HCV Abs +ve	4(5%)	16(5%)	**NS
Albumin < 35g/l	51(63%)	134(45%)	p- value 0.004, 95%CI 1.3-3.5

IQR- Interquartile range, HBsAg – hepatitis B surface antigen rapid test, HIV- HIV rapid serological test, Anti-HCV Abs- hepatitis C antibody test by rapid test, CI– confidence intervals

** NS - not significant.

One hundred and seventy nine (47%) patients had *De Ritis* ratio ≥2:1; whereas 35%, 58%, and 61% of all participants had an ALT, AST, and ALT or AST above 30IU/L respectively.

### Patient characteristics and alcoholic liver disease (ALD)

Thirty-nine subjects (10% of all study subjects and 48% of subjects with alcohol misuse) met the study definition for alcoholic liver disease. All participants with ALD had ALT less than 300IU/L. When compared to those without ALD, the thirty-nine subjects had a significantly greater proportion of male gender (65% of males compared to 43% females, p-value 0.011, 95%CI 1.2 to 4.8). Fifty four percent of subjects with ALD originated from the western (36%) and northern (18%) regions of Uganda, though the majority of subjects in the study (62%) originated from central region. Region of origin was significantly associated with ALD (p-value 0.001, 95%CI 0.1 to 0.3). Sixty one percent of those diagnosed with ALD were catholic, though type of religion did not vary significantly between those who were diagnosed with ALD compared to those without ALD (p-value 0.08, 95% CI -0.4 to 0.02). Fifty five percent of study participants with ALD had more than five sexual partners in their lifetime. Participants with ALD had more lifetime sexual partners than those without ALD (p- value 0.001, 95%CI 0.16 to 0.6).

Nearly half of our study participants were HIV sero-positive. Being HIV sero-positive did not vary significantly between those who had ALD and those who did not (54% versus 48 % respectively). Hepatitis B surface antigen by rapid test was positive in 15% of subjects with ALD and only 7% without a diagnosis of ALD. This difference was not significant (p-value 0.089, 95% CI -0.13- 1.8). Hepatitis C antibody by rapid test was prevalent at 5% in both diagnostic groups. Forty six percent of all participants had a serum albumin below 3.5g/dl. A significantly greater proportion of subjects with an albumin below 3.5mg/dl had ALD compared to those without ALD (67% vs. 47% p-value 0.02, 95%CI 0.12 to 1.5), (Table 2).

**Table 2:** Description and inference of clinical and laboratory characteristics of those with alcoholic liver disease compared to those without alcoholic liver disease

Variable	Alcoholic Liver Disease	No Alcoholic Liver Disease	Statistical Inference
N, proportion	39 (10%)	341 (90%)	
Median age (years)	35	35	
IQR (years)	29-45	26-50	
Male gender	25 (64%)	144 (42%)	p-value 0.009, 95%CI 1.2-4.9
**Region**			p-value 0.001, 95%CI 1.1-1.4
Central	15 (39%)	223 (65%)	
West	14 (36%)	46 (14%)	
North	7 (18%)	21 (6%)	
East	1 (3%)	33 (10%)	
Others	2 (5%)	18 (5%)	
**Religion**			p-value 0.08, 95%CI 0.7-1.0
Catholic	24 (61%)	116 (34%)	
Protestant	11 (28%)	111 (33%)	
Muslim	1 (3%)	71 (21%)	
Pentecostal	1 (3%)	30 (9%)	
Others	2 (5%)	13 (4%)	
**Number of lifetime sexual partners**			p-value 0.0006, 95%CI 1.2-1.8
1	4 (11%)	96 (32%)	
2	6 (15%)	53 (18%)	
3	4 (11%)	46 (15%)	
4	3 (8%)	16 (5%)	
5 or more	21 (55%)	91 (30%)	
HBsAg sero +ve	6 (15%)	25 (7%)	p-value 0.08, 95%CI 1.0-6.0
HIV sero +ve	21 (54%)	162 (48%)	**NS
AntiHCV Ab +ve	2 (5%)	18 (5%)	**NS
Albumin < 35 g/l	26 (67%)	159 (47%)	p-value 0.018, 95% CI 1.1-4.6

IQR- Interquartile range, HBsAg – hepatitis B surface antigen rapid test, HIV- HIV rapid serological test, Anti-HCV Abs- hepatitis C antibody test by rapid test, CI– confidence intervals

** NS - not significant.

**Figure 3:**
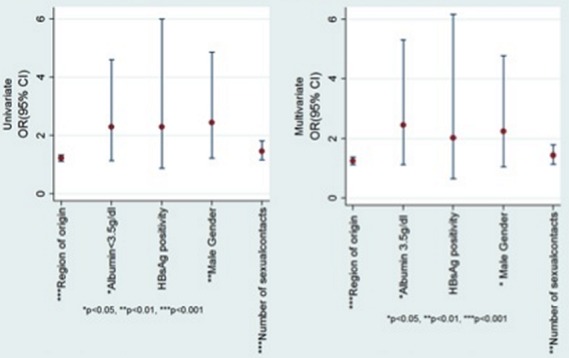
Analysis of ALD by variou sfactors - Graphic summary of p-values, odds ratios, and 95% confidence intervals for univariate and multivariate analysis for gender, region of origin, number of life time contacts, hepatitis B surface antigen status and serum albumin below 35g/l (3.5g/dl) among those with alcoholic liver disease compared to those without.

Multivariate analysis of factors that seemed to show a trend or definite association with ALD (male gender, number of life time sexual partners, region of origin, religion, positive HBsAg and serum albumin less than 3.5g/dl) on univariate analysis, revealed male gender (p-value 0.035, OR = 2.3, 95%CI 1.0-4.8), number of lifetime sexual partners (p-value 0.002, OR = 1.4, 95%CI 1.1-1.8), region of origin (p-value 0.001,OR = 1.3, 95%CI 1.1-1.4) and serum albumin below 3.5g/dl (p-value 0.023, OR = 2.5, 95%CI 1.1-5.3 ) was significantly associated with ALD (Table 2 and Figure 3)

## Discussion

Our study results show a high prevalence of alcohol use, misuse and ALD among patients presenting to the medical emergency admissions ward at the large urban hospital in Uganda. They also suggest alcohol misuse and ALD are more frequent among males, individuals with a greater number of lifetime sexual partners, and those originating from specific regions of Uganda.

Diagnosing alcohol misuse or ALD is a challenge in the emergency setting since patients often minimize/deny alcohol misuse and diagnostic tests for ALD are not very specific [5]. We used the CAGE because it has, within its limits, demonstrated reasonable accuracy and is easy to execute. CAGE measures indirectly the amount of alcohol intake, duration of intake and continuity of consumption that have all been implicated in the pathogenesis of ALD [11, 13]. A *De Ritis* ratio of ≥2:1 was used as a diagnostic marker of ALD since it has adequate accuracy and is more relevant in resource limited settings [12, 14, 15]. Moreover, this ratio has been found to indicate advanced ALD [16].

Alcohol use in our study is comparable to a previous International report from the Gender, Alcohol and Culture: An International Study (GENACIS) [8, 17]. The GENACIS report uncovered 47% of respondents reported alcohol use; this is exactly what we found in our study. Our findings of alcohol misuse are also similar to problem drinking among patients attending primary healthcare units in Kampala, Uganda [18] and to a prospective study in two European internal medicine departments that reported a prevalence of 19.6% and 20.5 % respectively [19]. We also found, like the GENACIS report that men are more likely to drink than females. The reasons for this gender preference have been discussed in the GENACIS report [8].

Like previous studies, our study shows a significant link between heavy drinking (12 or more drinks in a single day) or alcohol abuse and originating from the northern region of Uganda [8]. We found individuals from the north were twice as likely to be heavy drinkers compared to those from the central region. This can be explained by the civil strife that the region has experienced over the last twenty years [20]. What was new was hailing from the west of Uganda was also associated with alcohol use. The reason for this new finding is not clear.

Available studies have also reported an association between alcohol misuse and having a greater number of lifetime sexual partners, more so in male gender than in female gender [21]. This is akin to what we found in our study and might be a result of behavior modification following alcohol use.

The high HIV prevalence in our study population is not surprising. HIV and co-morbidities are still a major cause of adult medical admissions in our hospital regardless of HIV country prevalence to 7% [22]. In contrast to other published works, we did not find an association between HIV sero-positivity and alcohol misuse [21]. This could be explained by insufficient sample size and inadequate power to detect this association.

Overall, 10% of our study subjects met the study criteria for diagnosis of ALD, which is alarming. Few available published reports put the frequency of ALD between 3% and 10% among patients admitted to adult hospital care facilities [19, 23]. The prevalence in our study is at the high extreme and supports available data indicating high alcohol consumption in Uganda.

Use of liver enzymes for diagnosis of ALD is subject to confounding especially in Uganda where the national prevalence of chronic hepatitis B is 10% [5, 10, 24]. It could be argued that this may have lead to over diagnosis of ALD. However, no association between hepatitis B and ALD was found after correcting for other factors. Low serum albumin was associated with ALD; this has been described among patients with ALD [25, 26].

We admit that our study had limitations. At the outset, our study was a cross sectional study and carried with it all the limitations of such a study design [27]. It was not possible to quantify the amounts of alcohol consumed partly because available methods are inadequate in measuring the amount of traditional alcoholic beverages consumed in Uganda let alone estimating the concentration of alcohol in each beverage. We also recognize that this study is limited by the accuracy of the criteria we used for diagnosis of ALD.

## Conclusion

Overall, our study has demonstrated a high prevalence of alcohol misuse and ALD. In addition, male gender, region of origin, a greater number of sexual partners, and serum albumin below 3.5g/dl are associated with alcohol misuse and ALD. Like other studies, our study highlights the ongoing problem of alcohol misuse and alcoholic liver disease, and associated factors in this sub-Saharan African country.
